# A Microring Resonator Sensor for Sensitive Detection of 1,3,5-Trinitrotoluene (TNT)

**DOI:** 10.3390/s100706788

**Published:** 2010-07-13

**Authors:** Rozalia Orghici, Peter Lützow, Jörg Burgmeier, Jan Koch, Helmut Heidrich, Wolfgang Schade, Nina Welschoff, Siegfried Waldvogel

**Affiliations:** 1 Fraunhofer Heinrich-Hertz-Institute, Energy-Campus, Am Stollen 19, 38640 Goslar, Germany; E-Mails: peter.luetzow@hhi.fraunhofer.de (P.L.); helmut.heidrich@hhi.fraunhofer.de (H.H.); 2 LaserApplicationCenter, Clausthal University of Technology, Energy-Campus, Am Stollen 19, 38640 Goslar, Germany; E-Mails: j.burgmeier@pe.tu-clausthal.de (J.B.); jan.koch@tu-clausthal.de (J.K.); w.schade@pe.tu-clausthal.de (W.S.); 3 Kekulé Institute for Organic Chemistry and Biochemistry, University Bonn, Gerhard-Domagk-Str. 1, 53121 Bonn, Germany; E-Mails: nina.welschoff@uni-bonn.de (N.W.); waldvogel@uni-bonn.de (S.W.)

**Keywords:** integrated optics, ring resonator, optical waveguides, sensors, diode lasers, TNT

## Abstract

A microring resonator sensor device for sensitive detection of the explosive 1,3,5-trinitrotoluene (TNT) is presented. It is based on the combination of a silicon microring resonator and tailored receptor molecules.

## 1. Introduction

Detection of explosives is increasingly attracting more interest because of the global increase in terrorism. Highly selective and sensitive sensing techniques are required in order to detect trace explosives hidden on a person’s body or in baggage to provide security at airports, air travel and access control for sensitive infrastructures.

TNT is one of the major components of bulk explosives. It can also be used as a detonator for highly explosive shells or bombs. Due to its very low vapour pressure TNT is very difficult to detect in the gas phase [1].

Microring resonators have found numerous applications in chemical [2] and biochemical sensing applications [3–5]. The most important advantages of the microring resonator sensors in comparison to other TNT sensor devices, e.g., SPR sensors, are their operation for possible field applications, the high mechanical stability, scalability to sensor networks, and cost reduction due to wafer scale processing [6]. The detection principle is based on the accurate measurement of changes in the index of refraction of the receptor layer upon reversible complex formation in the presence of analytes (*i.e.*, TNT) covering the surface of a sensor device. This is done via a change of the resonance wavelength or intensity (at fixed wavelength).

While previous experimental studies on microring sensors were mostly restrained to measurements in solutions we demonstrate in this paper sensitive detection of TNT from the gas phase by using specially developed triphenylene-ketal based receptor molecules [7,8]. We believe that this sensor will be a key device for the selective and highly sensitive detection of TNT.

## 2. Sensing Principle

**Figure 1.**
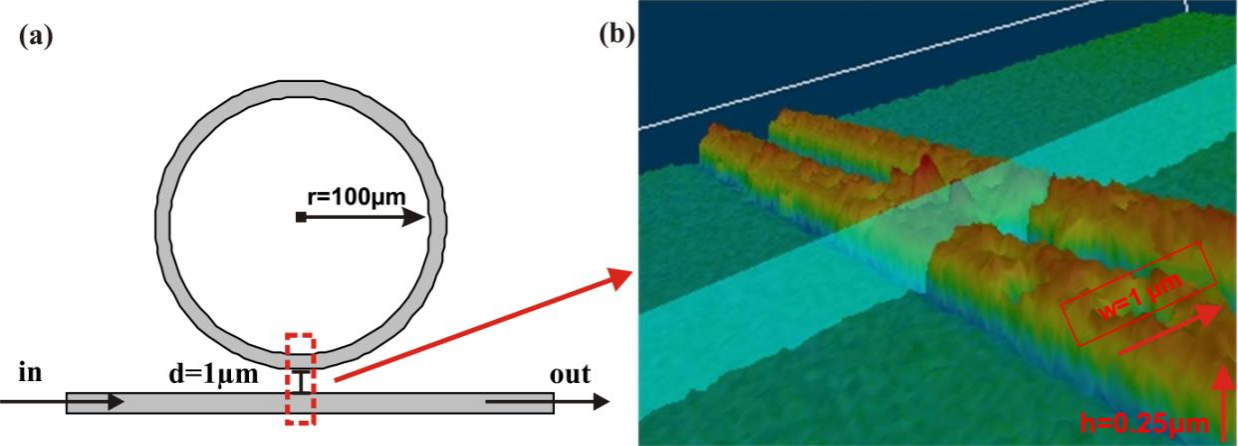
(a) Architecture of microring resonator; (b) microscope view of the coupling region.

The structure of a basic microring resonator is depicted in Figure 1a. A waveguide circuit with a radius of 100 μm is located in close vicinity of a 250 nm high and 1 μm wide straight waveguide forming a directional coupler. Figure 1b shows a microscope view of the coupling area between both waveguides. The straight waveguide serves as input and output waveguide, respectively. Light is coupled into the straight waveguide. Part of it couples into the ring resonator via evanescent coupling and then back into the straight waveguide also due to the evanescent coupling. On resonance of the microring the light that couples back into the waveguide is phase shifted by π resulting in destructive interference with the light that was not coupled into the ring leading to a reduction in output signal [9,10].

**Figure 2.**
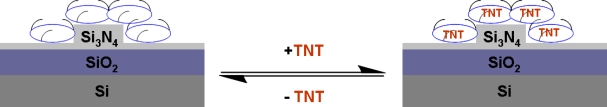
Schematic ridge structure of the microring resonator and reversible intercalation of TNT into schematically drawn receptors.

For the application as a TNT sensor, a silicon nitride microring resonator has been fabricated on a silicon substrate covered with approx. 5 μm of SiO_2_. The silicon dioxide assures optical isolation of waveguides from the silicon substrate (Figure 2).

A special receptor film based on triphenylene-ketal is used as cladding layer. The general synthesis of the receptor compounds is described in [7] and a detailed supramolecular study (solution experiments) about binding of explosives will be reported in due course. Surface coverage is performed by electrospray coating using a solution of receptor dissolved in tetrahydrofuran. The receptor molecules have the ability to selectively bind trinitrotoluene (TNT) molecules via a key-lock-principle. The supramolecular interaction between TNT and receptor molecules causes an intense colouring and consequently, also a change in the refractive index within the cladding layer which influences the light propagation within the microring resonator. A change of the refractive index of the cladding layer results in a change of the effective refractive index of the mode propagating within the ring resonator that can be observed by a shift of the resonance wavelength.

The resonance wavelength of the microring is given by the following equation:

$$m\cdot \lambda =n_{\mathrm{eff}}\cdot L$$

where *m* is the order of the resonant mode, *λ* is the resonance wavelength, *n_eff_* is the effective refractive index and *L* is the length of the ring resonator. By measuring the shift of resonance wavelength caused by changes in the effective refractive index due to the adsorption of the analytes, the detection of trinitrotoluene becomes possible.

## 3. Experimental

The experimental setup includes a tunable DFB laser diode with the central emission wavelength λ = 1.572 μm and typical spectral linewidth of 1 MHz. The laser diode is packaged in a 14-pin butterfly housing with built-in TEC, thermistor, optical isolator and polarization-maintaining fiber pigtail. The emission wavelength of the laser diode depends on its temperature and on the current; therefore, an accurate control of temperature and current is indispensable. For temperature stabilization a closed loop control algorithm has been developed. The current-control of the diode is done with a driver that has been developed in our working group. This driver allows the application of a voltage ramp in order to tune the wavelength linearly.

**Figure 3.**
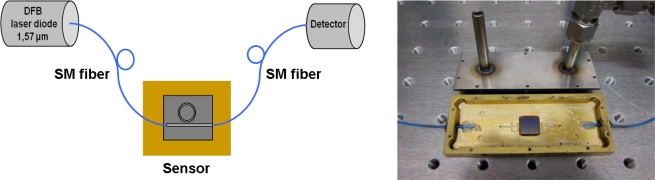
Schematic of the experimental setup and photo of the fiber coupled sensor.

The microring resonator sensor chip is connected to two single mode fibers which serve as input and output, respectively. This assembly is mounted in a brass cage that can simultaneously be used as cell for the on line measurements when the cover plate is modified and provided with two pipes, for injection of the analyte and exit, respectively (Figure 3). For measurement of the transmitted intensity an InGaAs photodiode is used. A schematic view of the experimental setup and a photo of the fiber coupled microring resonator sensor are shown in Figure 3.

The optical response of the microring resonators is highly sensitive to changes in the refractive index of the overlayer. For characterization of this sensor element the surface of the sensor chip has been coated with the receptor molecules via an established electrospray method [11,12]. The thickness d = 150 nm of the receptor film has been determined by on line measuring of the frequency shift of a quartz oscillator that is situated in close vicinity of the sensor chip.

Measurements with the microring resonator sensor have been done considering three explosive nitroaromatic compounds—1,3,5-trinitrotoluene (TNT), 2,4-dinitrotoluene (2,4-DNT), 1,3-dinitrobenzene (1,3-DNB)—and a non aromatic analyte—a solution of hydrogen peroxide (30% H_2_O_2_). The purchased high quality nitroaromatic analytes were recrystallized twice from methanol or ethanol. The identity was proved by matching their ^1^H-NMR spectra and melting points with literature values. The purity was determined by gas chromatography and only analyte samples with purity > 99.8% were employed for studies. The accuracy in purity is limited by base line separation in gas chromatography of potential impurities. The vapour pressures of the analytes at different temperatures are listed in Table 1. Due to the limited information regarding the vapour pressure of these analytes accessible in the literature, it was not possible to consider all vapor pressures at the same temperature. All measurements are done at room temperature (T_lab._= 21.8 °C); a vapour pressure of 5,29 ppb for TNT has been assumed.

For all measurements a gas mixing system consisting of two mass flow controllers has been used. This enables the injection of the analyte at given concentrations into the cell (sensor cage) by diluting the saturated vapour concentration of the analyte with a carrier gas (nitrogen). Therefore, small amounts of each analyte have been placed in separate closed round bottom flasks. When the saturated vapour concentration of the analyte was reached (after several hours), the round bottom flask has been connected to one mass flow controller in order to perform quantitative investigations regarding the sensitivity and selectivity of the microring resonator sensor. During the measurements the flow rate was 0.10 L/min. The flushing of the sensor with nitrogen for desorption is done with the same flow rate as well.

**Figure 4.**
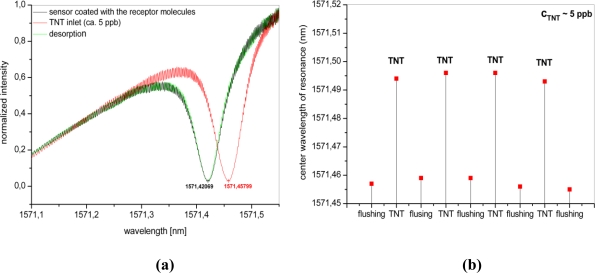
Wavelength shift of resonance measured after the inlet of TNT (a) and for several consecutive flows of TNT (b).

For TNT measurement the sensor has been flushed with nitrogen and a resonance spectrum of the microring resonator sensor coated with the receptor molecules was measured (Figure 4a). After flushing the sensor, TNT has been introduced into the cell and subsequently the sensor has been renewed flushed with nitrogen for decontamination. At resonance, light circulates many times within the ring and this causes an increase in the interaction length between the evanescent field and the receptor layer. With TNT adsorbed to the receptor molecules, a change in the effective refractive index occurs which is measured as a shift of the resonance peak (Figure 4a). When the sensor is flushed with the carrier gas for desorption, a return of the resonance to the initial position has been observed.

This measurement has been repeated for several times and nearly identical TNT flow concentrations in order to prove the reproducibility of the wavelength shift of the resonance for each inlet. Figure 4b shows the positions of the resonance after flushing with nitrogen and after enrichment with TNT molecules.

**Figure 5.**
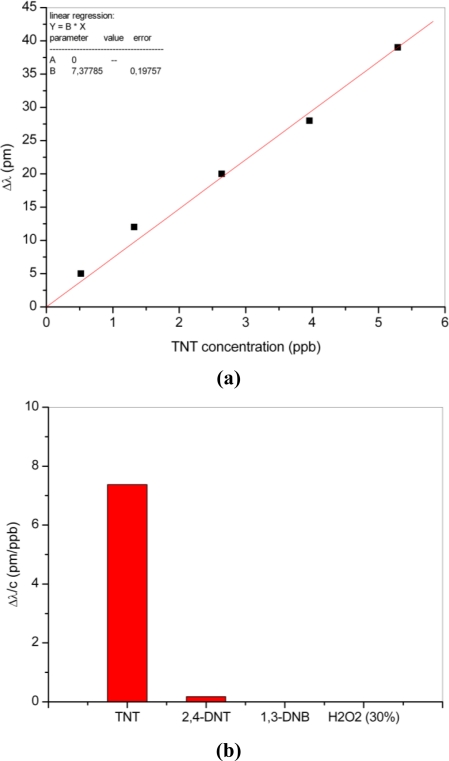
Wavelength shift of resonance measured for different concentrations of TNT (a); response of the sensor to TNT and other potentially interfering analytes (b).

The introduction of the TNT containing flow into the sensor cell is done at room temperature and its concentration can be accurately controlled by adjusting the flow of the carrier gas through the TNT recipient. Further measurements have been performed with TNT by diluting the saturated vapor concentration (5,29 ppb) from 100% to 10% with the carrier gas. The linear dependence between the TNT concentration and the wavelength shift of the resonance is shown in Figure 5a.

In order to prove the response of the sensor to interaction with other molecules, e.g., the nitroaromatic compounds, measurements with 2,4-DNT, 1,3-DNB and the non aromatic H_2_O_2_-solution have been performed. For this investigation the wavelength shift of the resonance has been measured for different concentrations of the analyte, from 100% to 10% of the equilibrium vapour pressure, respectively. From the slope of the linear regression a sensitivity of the microring resonator sensor to each analyte can be calculated. Figure 5b shows the shift of the resonance wavelength in [pm/ppb] for each investigated analyte. As can be seen, the sensor shows high sensitivity in the presence of TNT molecules and no response behaviour to 1,3-DNB and H_2_O_2_. The low sensitivity of the sensor to 2,4-DNT molecules is caused by the similar structure of these molecules to the TNT molecules. This similarity enables the partial attachment of the molecules to the receptor molecules.

## 4. Conclusions

Miniaturized evanescent field sensor elements based on microring resonators are gaining recently much attention due to their high sensitivity. In this paper the functionality of a microring resonator as sensor device for the detection of the explosive TNT has been proven. First measurements with a fiber coupled sensor chip are shown and discussed. In addition, measurements with other analytes (2,4-DNT, 1,3-DNB and 30% H_2_O_2_) have been performed to demonstrate the selectivity which can be obtained by coating the microring resonator with specially developed receptor molecules for reversible and selective trapping of TNT molecules.

Next steps that will be pursued are further investigations on the selectivity of this novel approach for sensing the explosive TNT, the optimisation of the thickness of the receptor film in order to achieve a higher sensitivity and selectivity of the sensor and the performance of differential measurements in a setup composed of two or more microring resonators located close to a common straight waveguide.

## Figures and Tables

**Table 1.** Vapour pressure of TNT, 2,4-DNT, 1,3-DNB and H_2_O_2_ (30%).

	Vapour Pressure (ppb)	Temperature (°C)	References
TNT	5,29 7,7	25 25	[13] [14]
2,4-DNT	313	21.8	[15]
1,3-DNB	5720	25	[16]
H_2_O_2_ (30%)	227*10^5^	20	[17]
